# The Effects of *Paracoccidioides brasiliensis* Infection on GM-CSF- and M-CSF-Induced Mouse Bone Marrow-Derived Macrophage from Resistant and Susceptible Mice Strains

**DOI:** 10.1155/2015/605450

**Published:** 2015-10-12

**Authors:** Calliandra de Souza Silva, Aldo Henrique Tavares, Marcio Sousa Jeronimo, Yasmin Soares de Lima, Lorena da Silveira Derengowski, Anamélia Lorenzetti Bocca, Ildinete Silva-Pereira

**Affiliations:** ^1^Laboratório de Biologia Molecular, CEL/IB, Campus Darcy Ribeiro, Asa Norte, 70910-900 Brasília, DF, Brazil; ^2^Laboratório de Imunologia Aplicada, Departamento de Biologia Celular, Instituto de Biologia, Universidade de Brasília, Campus Universitário Darcy Ribeiro, 70910-900 Brasília, DF, Brazil

## Abstract

Considering the importance of macrophages as the first line of defense against fungal infection and the different roles played by the two M1- and M2-like polarized macrophages, we decided to evaluate the effects of *Paracoccidioides brasiliensis* infection on GM-CSF- and M-CSF-induced bone marrow-derived macrophages (BMM) from the A/J and B10.A mouse strains, an established model of resistance/susceptibility to PCM, respectively. Upon differentiation, the generated GM- or M-BMMs were characterized by morphological analyses, gene expression profiles, and cytokines production. Our main results demonstrate that GM-BMMs derived from A/J and B.10 produced high levels of pro- and anti-inflammatory cytokines that may contribute to generate an unbalanced early immune response. In accordance with the literature, the B10.A susceptible mice lineage has an innate tendency to polarize into M1-like phenotype, whereas the opposite phenotype occurs in A/J resistance mice. In this context, our data support that susceptibility and resistance are strongly correlated with M1 and M2 polarization, respectively.

## 1. Introduction

The increased incidence of fungal diseases has been ascribed to the rise in both the number of immunocompromised patients [1–3] and the number of cases in so-called immunocompetent individuals [4–8]. These data indicate that fungal infections are a worldwide health problem with high rates of mortality and morbidity. In Brazil, the situation is not very different, since, between 1996 and 2006, about 3,583 deaths occurred as a result of fungal diseases [9, 10], with Paracoccidioidomycosis (PCM) being the most common systemic mycosis not only in Brazil but also in Latin America [11]. This number may be even higher considering that the notification of patients diagnosed with systemic mycosis is not mandatory [12].

A murine model of resistance/susceptibility to PCM has been established, in which isogenic B10.A mice develop an immune response analogous to a susceptible human host, and the isogenic A/Sn or A/J strain develops a response equivalent to a resistant host [11, 13–16]. The resistance to PCM in both human and murine hosts is associated to a more efficient cell mediated immune response and activation of phagocytes throughout the infection. Although there is no classic Th1/Th2 polarization response, the secretion of IL-12 and IFN*γ* has been demonstrated as protective [11, 17–19]. The controlled progression of the disease is associated with an initially slower proinflammatory immune response, which further allows the development of a more robust resistance pattern during the course of infection [20]. In contrast, the susceptibility is associated to a decreased immune cellular response due to premature deactivation of T-cell mediated immunity and preferential B-cell activation, in addition to increased levels of IL-10 or TGF-*β* [11, 17]. Its progression is connected to a more efficient initial proinflammatory immune response, which is later downregulated [20]. In both cases, macrophages play a crucial role in the regulation of fungal growth in the early stages of infection [13]. Recently, it has been shown that the cytokine IL-17 plays a key role in innate antifungal defence, contributing to fungal clearance as observed in the best studied model of mucosal candidiasis (revised in [21]). During* P. brasiliensis* infection, the production of IL-17 was observed and its level was increased in response to the absence of TLR-2 activation and an uncontrolled inflammatory response with low number of regulatory CD4^+^CD25^+^FoxP3^+^ T-cells was observed. However, the survival time was not affected by the presence or the absence of TLR-2 [22].

Macrophages are known for their role in initiating and directing immune responses* in vivo*. Although there have been arguments about the spectrum to which a macrophage can be activated, differentiated macrophages are usually divided into two major groups, M1/classically activated macrophages and M2/alternatively activated macrophages [23, 24]. In general, M1 cells have IL-12^high^, IL-23^high^, and IL-10^low^ phenotype. On the other hand, the various forms of M2 (M2a, M2b, M2c, and M2d TAM) cells share IL-12^low^, IL-23^low^, and IL-10^high^ phenotype [24]. This functional and reversible plasticity is dependent on the activation state, which is primed by particular signals specific to tissues and local microenvironments [23, 25]. In this regard, there are compelling evidences indicating that, according to how, when, and the type of differentiation conditions, the phenotypes M1-M2 will determine the multifactorial outcomes in immune response. In* Cryptococcus neoformans* pulmonary infection, the polarization status changes over time due to either repolarization of individual macrophages or replacement of M2-polarized (nonprotective) by new M1-polarized (protective) cells [26]. Davis et al. [27] demonstrated* in vitro *that, independent of any previous stimulation, macrophage polarization is “phenotypically and functionally plastic in response to changing cytokine and fungus-sensing environments,” with the final stimulus determining the fungicidal potential. Macrophage plasticity is probably the mechanism used by* Candida albicans* to increase pathogenicity/survival, by changing environmental cues that induce M2 to M1 switch [28, 29]. The therapeutic repolarization of macrophages may open the door to interventions that could be useful in the treatment of fungal diseases [27].

In PCM, alveolar macrophages are probably one of the first immune cells to interact with* P. brasiliensis.* The results of this interaction, associated with the host health, other signals (e.g., damage-associated molecular pattern molecules, DAMPs), and genetic background will determine the infection outcome. It has been shown that this fungus is phagocytized by macrophages both* in vivo* and* in vitro*, but only properly activated macrophages manage to become fungicidal [30]. Recently, Feriotti et al. [31] demonstrated that when peritoneal macrophages from resistant (A/J) and susceptible (B10.A) mice were exposed to* P. brasiliensis*, they exhibit increased expression of “M1-like” (iNOS and SOCS3) and “M2-like” (Arginase-1, FiZZ1, YM1, and SOCS1) differentiation markers, respectively. Indeed, and in accordance with related articles, it seems that the apparent susceptibility to* P. brasiliensis *is associated with an exacerbated initial innate immune response, mediated by classically activated macrophages (M1-like) and a lack of fungal growth control. On the other hand, resistance is associated with an early moderated proinflammatory response, mediated by alternatively activated macrophages, evolving to a better control of fungal burden in the later stages of infection. However, these M2 macrophages did not show the classical differentiation markers [31].

To better understand the role of M1/M2-like macrophage in the PCM murine model, in this work, we evaluated phagocytic and secretory abilities, as well as expression analyses of some genes related to mice antifungal responses in GM-CSF (M1-like) and M-CSF (M2-like) induced bone marrow macrophages obtained from of A/J and B10.A mouse strains infected* in vitro* with a virulent strain of* P. brasiliensis*.

## 2. Materials and Methods

### 2.1. Fungus and Culture Conditions

The virulent strain Pb18 of* P. brasiliensis* was maintained by weekly subcultivation in semisolid Fava Netto's medium at 36.5°C and was used in the experiments after 7 days of growth. Yeast cells were resuspended and adjusted to the desired concentration based on hemocytometer counts using the Janus Green B vital dye to determine viability [32]. The fungal viability used in these tests was always higher than 90%. The virulence of the strain was maintained by* in vivo* passages in mice every 3 months.

### 2.2. Mice

*P. brasiliensis* resistant (A/J) and susceptible (B10.A) strains of 6 to 12 weeks old male mice [14, 16, 20, 33] were obtained from the Immunology Department of the University of São Paulo Biomedical Sciences Institute, Brazil. The animals were housed with food and water* ad libitum* at the Animal Care Center of the Biological Institute of the University of Brasilia, Brazil. The mice were euthanized in a carbon dioxide chamber, and their bone marrows were collected. All procedures involving animal were performed following the guidelines for the use of animals according to Brazilian laws and were approved by the Committee of Ethical Use of Animals (Proc. UnBDoc 52657/2011).

### 2.3. *Ex Vivo* Infection of GM-BMM (GM-CSF-Induced Bone Marrow-Derived Macrophage) and M-BMM (M-CSF-Induced) Cells from* P. brasiliensis* Resistant and Susceptible Mouse Strains

The two different BMMs populations were obtained using recombinant GM-CSF (20 ng/mL PeproTech) or M-CSF (30% (v/v) of L929 cell-conditioned medium) according to Tadokoro and de Almeida Abrahamsohn [34]. In this work, the nomenclature adopted is GM-BMM or M-BMM, related to GM-CSF- or M-CSF-induced macrophage differentiation condition, respectively. Briefly, the isolated BMMs from each mouse strain were cultivated in RMPI 1640 medium with 10% fetal bovine serum containing GM-CSF and M-CSF for 7 days when the adherent cells were recovered [34]. These cells were infected or not with* P. brasiliensis* at a cell-to-yeast ratio of 5 : 1 (multiplicity of infection, MOI: 5 : 1) for 6 h (transcription and ELISA assays) or 24 h (ELISA assays) in a humidified atmosphere of 5% CO_2_ at 37°C. This MOI has been previously shown to be nondeleterious to macrophage cultures [35, 36].

### 2.4. Ratio of Internalized/Adhered* P. brasiliensis* Yeast Cells by the Two Different BMMs of A/J and B10.A Mouse Strains

After 6 or 24 h of infection, adherent macrophages were washed with medium at 37°C, fixed, and stained with Panotic Staining kit. The number of macrophages with phagocytized or adhered yeasts was recorded by optical microscopy from a total of 300 cells to determine the percentage of adhered/internalized* P. brasiliensis* yeast cells. The experiments were performed in triplicate and five to ten microscopic fields were analyzed.

### 2.5. Cytokines and Chemokine Measurements

The cytokines TNF-*α*, IL1-*β*, IL6, and IL10 and the chemokine MCP-1 levels present in cell culture supernatants were measured by a capture enzyme-linked immunosorbent assay (ELISA) using the specific kits from eBioscience, according to the manufacturer's instructions. The absorbance values were measured in spectrophotometer (SpectraMax M5, Molecular Devices) and analyzed with SoftMax 5.2 software. Cytokines and chemokine concentrations were determined using a standard curve, following the kit recommendations. All determinations were performed in triplicate.

### 2.6. Quantitative Real-Time PCR (qRT-PCR) and PCRarray

The total RNA of the cultured macrophages was obtained employing the RNAeasy Plus Mini Kit (QIAGEN cat. number 74134), in accordance with the manufacturer's protocol. After DNase I treatment (included in the RNeasy Mini Kit Plus), first-strand cDNAs were synthesized from 500 ng of total RNA for each sample following the instructions of SuperScript III (Invitrogen). To confirm the GM- and M-CSF phenotype, the expression of markers genes was tested using specific primers for iNOS (forward-CGAAACGCTTCACTTCCAA, reverse-TGAGCCTATATTGCTGTGGCT) and Arginase-1 (forward-GTTCCCAGATGTACCAGGATTC, reverse-CGATGTCTTTGGCAGATATGC). The internal control used was 40S ribosomal protein S9 (RPS9) gene (forward-CGCCAGAAGCTGGGTTTGT, reverse-CGAGACGCGACTTCTCGAA) [36]. The qRT-PCR was performed using SyBr Green Master Mix (Applied Biosystems) with the standard cycling condition for this dye.

For the PCR array, after quantitative and qualitative analysis of total RNA, 1 *μ*g was reversely transcribed to cDNA using the RT^2^ First-Strand Kit (SA Biosciences), according to manufacturer's protocol. Subsequently, the cDNA samples were labeled with RT^2^ Real-Time SYBR Green PCR Master Mix (SA Biosciences) and added to 96-well plates of Mouse Antifungal Response RTC Profiler PCR Array (PAMM 00147Z, SA Biosciences/Quiagen). This array profiles the transcriptional levels of 84 critical genes involved in the innate immune response to fungal pathogens. These genes encompass those related to fungal pattern recognition receptors (PRRs) and their associated signal transduction, inflammation, and phagocytosis. In addition, 5 housekeeping genes for normalization of the PCR data and controls for genomic DNA contamination, reverse transcription efficiency, and PCR performance are included on each array. In our experimental conditions, two housekeeping genes (B2M and GUSB) had constant mRNA levels between control and experimental group and were used for data normalization. Product amplification, data acquisition (obtained as threshold cycle (Ct) values), and melting curve were performed by the ABI 7500 qRT-PCR system (Applied Biosystems, software version 2.0.3). Fold differences in gene expression between control and experimental groups were determined using the comparative threshold method (2^−ΔΔCt^ algorithm) [37]. Genes significantly modulated were identified based on the following two criteria: (i) the fold difference in average 2^−ΔΔCt^ values was greater than 2 or less than −2 (indicative of upregulation or downregulation, resp.) and (ii) the difference of the replicate 2^−ΔΔCt^ values for each gene in the control group and treatment groups was statistically significant (*p* < 0.05) according to Student's *t*-test between control and experimental groups. Data were analyzed by RT^2^ PCR Array Data Analyses profile version 3.5 available at http://pcrdataanalysis.sabiosciences.com/pcr/arrayanalysis.php.

### 2.7. Statistical Analysis

The differences between the groups were analyzed by Student's *t*-test or by two-way ANOVA with Turkey's multiple comparisons posttest performed using GraphPad Prism Mac 6.0d, GraphPad Software, La Jolla California USA, http://www.graphpad.com/. A *p* ≤ 0.05 was considered significant.

## 3. Results and Discussion

### 3.1. Characterization of GM-BMM and M-BMM of A/J and B10.A Mice Strains

Our group is interested in studying the host innate immune responses to pathogenic fungi, with emphasis on the role of macrophages from A/J e B10.A mouse strains, which is a well-established model of resistance/susceptibility to PCM [14, 16]. Considering the importance of these phagocytic cells as the first line of defense against fungal infection and the different roles played by the two subtypes of polarized macrophages, we decided to evaluate the response of GM- and M-CSF-induced BMMs of A/J and B10.A mouse strains infected with* P. brasiliensis*. Upon differentiation, the generated GM- or M-BMMs were characterized by morphological analyses and by their gene expression profiles, described as important markers to each polarized macrophage subtype.

**Figure 1:**
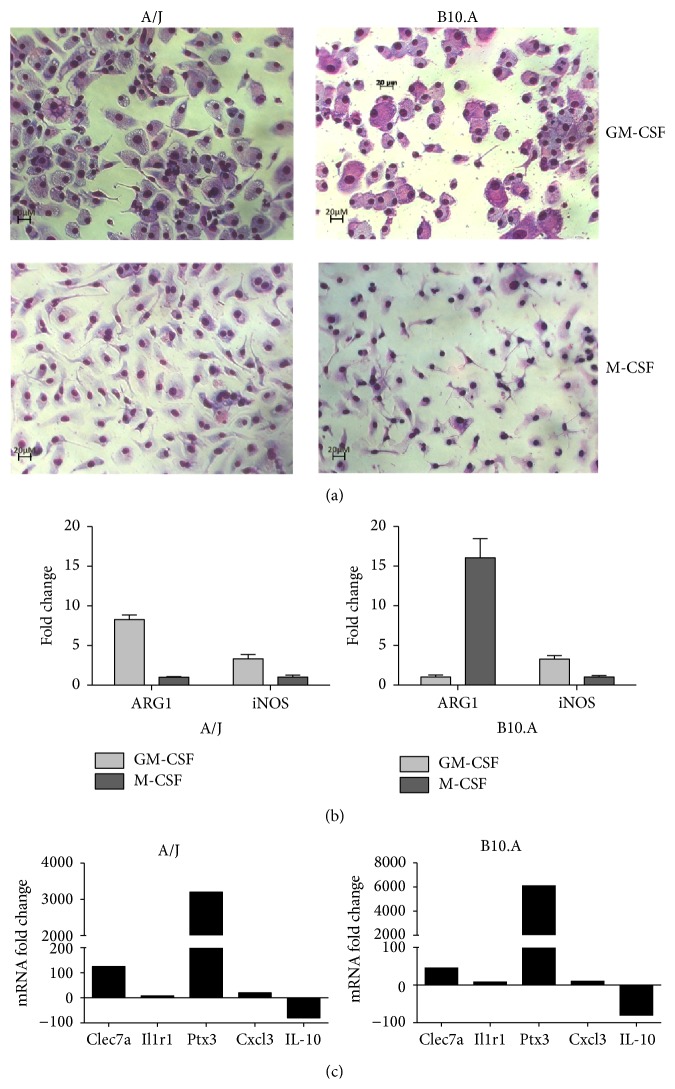
Characterization of GM-BMM and M-BMM of A/J and B10.A mice. Murine bone marrow cells were differentiated into macrophages (BMM) in the presence of GM-CSF (GM-BMM) or M-CSF (M-BMM) as described in Section 2. (a) Photomicrography of GM-BMM and M-BMM from A/J and B10.A mouse strains stained with Panotic kit (×200). (b) Quantitative PCR analysis (qRT-PCR) of induced nitric oxide synthase (iNOS) or Arginase-1 (ARG-1) mRNA expression from GM-BMM or M-BMM. Bars with SD represent the mean of fold change of the gene expression and are shown as *n*-fold difference of GM-BMM to the M-BMM cells. Fold change values were determined after each gene was normalized to the constitutively expressed rps9 gene. Data is representative of three separate experiments. (c) qRT-PCR analysis of Clec7a, Ilr1, Ptx3, Cxcl3, or Il10 mRNA expression from GM-BMM or M-BMM. Bars represent the mean of fold gene expression and are shown as *n*-fold difference of GM-BMM to the M-BMM cells. Fold change values were determined after each gene was normalized to the constitutively expressed Gusb (A/J) and B2m (B10.A) genes using the comparative threshold method.

A similar morphology was observed in the GM-BMM from A/J and B10.A mice. Both cell subtypes revealed an abundant and slightly acid granular cytoplasm, filled with vesicles/vacuoles (Figure 1(a)). This feature was also described by McWhorter et al. [38], who observed that M1-polarizing stimuli (LPS+IFN*γ*) caused cells to flatten into a round, pancake-like shape. Another aspect in common was related to their tendency to form multinucleated giant cells, similar to those found in several pathological conditions, including the foreign body response and infection sites of tuberculosis and cryptococcosis, which were described* in vitro* with human monocytes [39, 40] (data not shown). Conversely, M-BMM from the two mouse strains showed a reduced, smooth, and basophilic cytoplasm (Figure 1(a)). Again, this elongated cell morphology was similar to that observed with monocytes exposed to M2-polarizing stimuli (IL-4+IL-13), as also described by McWhorter et al. [38]. These macrophages also tend to form cell aggregates around* P. brasiliensis* yeasts cells (data not shown). Although the GM-BMM and M-BMM revealed morphological resemblances in the two mouse strains, the expression profiles of Arginase-1 (Arg-1) and induced Nitric Oxide synthase (iNOS) marker genes were different.

The* in vitro* expression profile of these marker genes is well known from studies with typical polarized M1 and M2 macrophages stimulated with IFN-*γ* and IL-4, respectively, which resulted in iNOS^high^ and Arg-1^low^ in M1 and iNOS^low^ and Arg-1^high^ in M2 [23, 41]. In the present work, the culture conditions used to obtain the different types of BMMs employed only GM-CSF or M-CSF, without any other postdifferentiation mediator. The analysis of the expression profiles revealed that only the BMMs of B10.A strain developed the expected pattern (iNOS^high^ and Arg-1^low^ in response to GM-CSF and iNOS^low^ and Arg-1^high^ to M-CSF stimulus), as shown in Figure 1(b). A different expression pattern was observed with GM-BMM of A/J strain, revealing a discrete induction of iNOS, a slight upregulation of Arg-1, and a lack of induction of both genes in M-BMM (Figure 1(b)). The paucity of data related to marker genes expression profiles of* in vitro* polarized BMMs from A/J and B10.A mouse strains causes some difficulty in exploring our data. Altogether, the culture conditions and the different mouse strains could explain the absence of a clear expression profile of the marker genes when compared to* in vitro* IFN-*γ* and IL-4 poststimulated M1 and M2 macrophages, respectively. Of note, several other genes besides iNOS and Arg-1 are differently expressed in GM-BMM and M-BMM cells. In fact, Lacey et al. [42] using a whole murine genome microarray showed 4206 genes differentially regulated between the murine C57BL/6 GM-BMM and M-BMM, with no poststimulation, including Clec7a, Il1r1, Ptx3, Cxcl3 (upregulated), and IL-10 (downregulated). As shown in Figure 1(c), these genes showed the same transcriptional pattern regardless of the mice strain, when we compared GM-BMM versus M-BMM. In sum, these results suggest a polarized differentiation in our cells.

**Figure 2:**
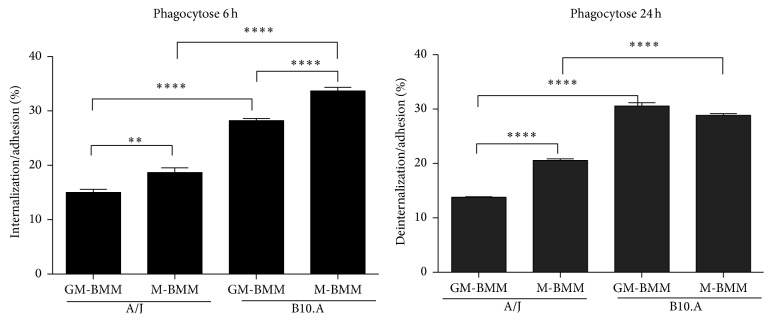
Ratio of internalized/adhered* P. brasiliensis* yeast cell by GM-BMMs and M-BMMs of A/J and B10.A mouse strains. Phagocytosis assays were performed employing a MOI (multiplicity of infection) of 5 : 1 macrophage to* P. brasiliensis* (Pb18) yeast cells, for incubation times of 6 h and 24 h. After the coculture, the cells were stained with Panotic kit. An average of 300 macrophages was counted and the number of ingested and/or adherent yeasts was determined. (^*∗∗∗∗*^ *p* ≤ 0.0001, ^*∗∗∗*^ *p* ≤ 0.001, ^*∗∗*^ *p* ≤ 0.01, and ^*∗*^ *p* ≤ 0.05).

To comparatively evaluate the initial interaction between the two types of BMMs, from both susceptible/resistant mouse strains and* P. brasiliensis* yeast cells, we determined the number of adhered/internalized fungal cells by the different BMMs. Since the formation of multinucleated giant cells observed in GM-BMMs and cells aggregates in M-BMM made counting individual macrophages difficult, the percentage of yeast cells internalized/adhered only took into consideration individual infected/noninfected macrophages. After 6 h of infection, regardless of the differentiated macrophage type, BMMs derived from B10.A strain showed higher percentage of internalization/adhesion than A/J strain (Figure 2). This agrees with the hypothesis that the susceptibility to* P. brasiliensis *is associated with an exacerbated initial innate immune response, while resistance is associated with an initially moderate response [11, 20]. Furthermore, as shown in Figure 2, a significantly higher percentage of internalized/adhered yeast cells was observed in M-BMMs when compared to GM-BMMs in both mouse strains (to both AJ and B10.A, M-BMM > GM-BMM). After 24 h of coculture, the same pattern was observed, with significantly higher percentage of internalization/adhesion in B10.A than A/J BMMs. In addition, despite being higher than A/J BMMs, both BMMs from B10.A presented relatively similar results (GM-BMM similar to M-BMM), while in A/J there was a significantly higher percentage of internalization/adhesion in M-BMMs than GM-BMMs (M-BMM > GM-BMM).

## 4. Gene Expression Profiling of GM- and M-BMMs from A/J and B10.A Mice Infected with* Paracoccidioides brasiliensis *and Cytokines Production

**Figure 3:**
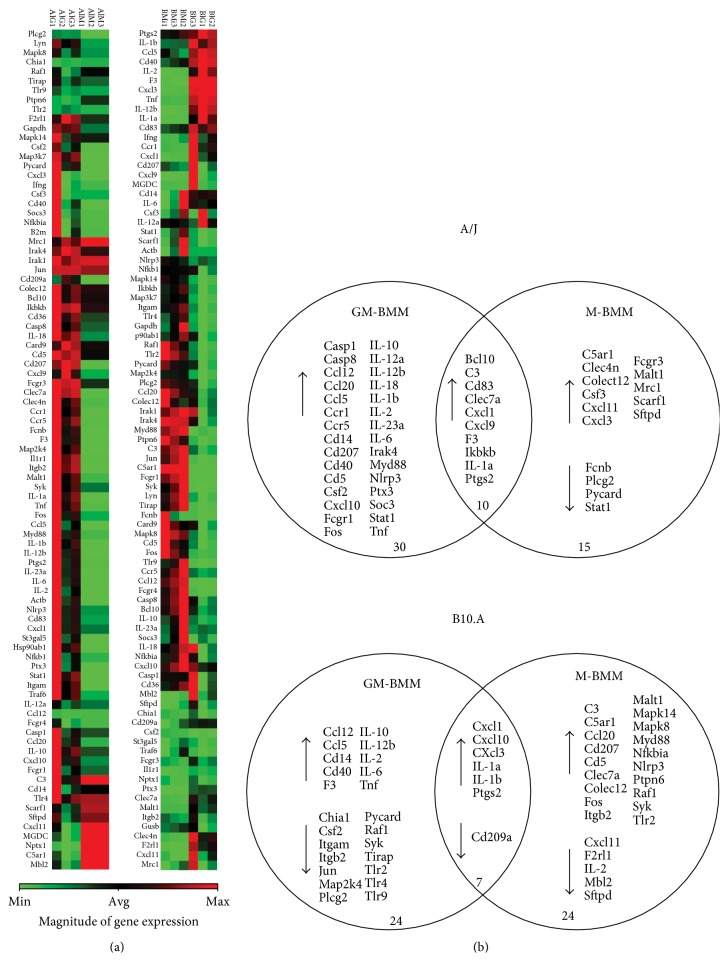
Expression profiling based on Mouse Antifungal Response RTC Profiler PCR Array. (a) A heat map was generated with 84 genes associated with antifungal immune response and five housekeeping genes, using RT^2^ Profiler Data Analysis Software version 3.5 with the PAMM 00147Z array panel (SABiosciences). Fold change values were determined after each gene was normalized to the constitutively expressed Gusb (A/J) and B2m (B10.A) genes using the comparative threshold method. AIGs 1, 2, and 3 and AIMs 1, 2, and 3 stand for A/J GM-BMM and M-BMM, respectively, infected with* P. brasiliensis *in relation to its respective control cells. BIGs 1, 2, and 3 and BIMs 1, 2, and 3 stand for B10.A GM-BMM and M-BMM, respectively, infected with* P. brasiliensis *in relation to its respective control cells. (b) Venn diagram summarizing the results of differentially expressed genes (*p* ≤ 0.05; fold change ≥2) between the noninfected and* P. brasiliensis *infected group of GM- or M-BMM of A/J and B10.A mice strains.

The pattern of gene expression in GM- and M-BMMs from A/J and B10.A mice infected with* P. brasiliensis* yeast cells was assessed using the Antifungal Response RT^2^ Profiler PCR Array (Quiagen). As described in the methodology, this array profiles the transcriptional levels of 84 critical genes, classified in important functional categories of the innate immune response to fungal pathogens.  Figure 3(a) shows the gene expression heat map of all 84 genes in GM- and M-BMMs from both mice strains infected with* P. brasiliensis*. Specifically, in GM- and M-BMMs from A/J mice, fungal infection results in a significant transcriptional modulation (up- or downregulation) of 55 genes. Among them, 10 genes were similarly induced in both BMMs, 30 exclusively in GM-BMM, and 15 in M-BMM. Similarly, in GM- and M-BMMs from B10.A mice, 7 genes were commonly modulated, 24 were exclusively in GM-BMM, and 24 were in M-BMM (Figure 3(b)). Based on the findings of previous fungal-phagocyte interaction studies, we selected modulated genes and clustered them into different functional categories as shown in Table 1.

Activation of macrophages is one of the first events in the innate immune response to fungal infections. This activation occurs upon recognition of conserved components of fungal cells by germline-encoded pattern recognition receptors (PRRs) of the phagocytic innate immune cells. Accordingly, several PRRs and PRRs signal transduction-encoding genes were modulated in BMMs infected with* P. brasiliensis*. Toll-like receptors (TLR) and C-lectin type receptors (CLR) are considered as being the main PRRs families involved in fungal recognition [21].

Our PCRarray data shows that the infection of GM- and M-BMMs from A/J mice with* P. brasiliensis* leads to no modulation of TLR2, TLR4, and TLR9 coding genes. Conversely, GM-BMM from B10.A had significant lower transcript levels of these TLRs, whereas TLR2 and MYD88 (protein adaptors that are critically important to TLR signaling pathway) were induced in M-BMM. Interestingly, expression of the gene coding TLR2 increased in dendritic cells after infection of susceptible mice with* P. brasiliensis*, but not in the resistant ones [43]. Furthermore, [22] demonstrated* in vitro* and* in vivo* that the lack of TLR2 usage and signaling resulted in a less-severe fungal infection, despite the fact that TLR2 deficient and normal mice showed equivalent survival times. Thus, TLR2 engagement could be employed as an evasion mechanism of* P. brasiliensis *and other PRRs may play an important role in the host immune response against* P. brasiliensis* infection.

Another important PRR to* P. brasiliensis* recognition and cellular activation is Dectin-1 [44]. According to these authors, the absence of Dectin-1 receptor drives macrophages to M2 phenotype with an anti-inflammatory activity that results in a lower nitric oxide production and an increased fungal growth [44]. Our results showed that Dectin-1 and Dectin-2 genes were upregulated in both BMMs of A/J (Table 1). The Dectin-1 gene was also upregulated in M-BMM of B10.A but was nonmodulated in GM-BMM, while Dectin-2 gene was nonmodulated in BMMs of B10.A. The importance of Dectin-1 in antifungal defense is well established in several fungi as* Candida *sp.,* Aspergillus* sp., capsule deficient* C. neoformans,* and* Histoplasma capsulatum *[45, 46]. Recently, it has been shown that Dectin-1 and Dectin-2 receptors were involved in the interaction with* Fonsecaea pedrosoi* spores, while spore recognition by Dectin-2 was responsible for the development of antigen-specific Th17 response [47].

The Mannose receptor (MR) gene was upregulated in GM-BMM of A/J; however, it was downregulated in both macrophages from B10.A. Feriotti et al. [31] showed that* P. brasiliensis* infection of a murine strain where MR was blocked by monoclonal antibodies resulted in a decrease of macrophage killing abilities as well as nitric oxide production. Moreover, the authors using flow cytometry assay have shown that* P. brasiliensis* induced a lower expression of this receptor on A/J macrophages, different to what we observed here, by transcript level analysis. The MR engagement was associated with classical macrophage activation and susceptibility of B10.A strain [31]. However, this apparent conflicting result may be probably due to a very poor correlation observed for comparisons between mRNA and protein levels [48, 49].

The downstream CLR signaling is important for the generation of an efficient cellular activation and fungal elimination. Dectin-1 transduces downstream signaling via Src and Syk family kinases and/or by a second pathway via Raf. These pathways lead to cytokine genes transcription and protein secretion [45, 46]. The Syk and Raf-1 genes were nonmodulated in GM- and M-BMM from A/J (Table 1), while they were upregulated in M-BMM and downregulated in GM-BMM from B10.A. Following Dectin-1 activation, the intracellular Raf-1 and Syk-dependent signaling pathway was demonstrated crucial for fine-tuning cytokine gene expression [50].

According to Feriotti et al. [31], A/J mice experimentally infected with* P. brasiliensis* showed M2-like differentiation macrophages, while B10.A mice showed M1-like differentiation macrophages. Analyzing the gene expression profile of GM-BMM from B10.A and M-BMM from A/J, the B10.A macrophages showed a decreased gene expression of CLR signaling molecules that could impair the efficient cell activation and, despite their M1-like phenotype, the control of antifungal defense seems not to be correctly activated.

Another crucial aspect of the innate immunity against fungal pathogen is the activation of the inflammasome, a cytoplasmic protein complex composed of PRRs such as NLRP3 (NOD-like receptor family, pyrin domain-containing 3), an adaptor protein ASC (apoptosis-associated speck-like protein containing a CARD), and procaspase-1. Upon formation of the complex, procaspase-1 is cleaved into an active cysteine protease, which further leads to the maturation of the proinflammatory cytokines IL-1*β* and IL-18. Recently, we have shown that* P. brasiliensis* activates NLRP3 inflammasome [51]. Feriotti et al. [31] found that macrophages from the susceptible B10.A mice infected with* P. brasiliensis *exhibited the typical markers of a proinflammatory “M1-like” (GM-BMM) differentiation. In contrast, A/J macrophages exhibited an alternatively activated “M2-like” (M-BMM) differentiation. In this context, here is shown that B10.A macrophages, regardless of M- or GM-CSF-induced differentiation, showed high IL-1*β* transcript accumulation, suggesting an “M1-like” differentiation pattern, whereas in M-BMM of A/J, neither NLRP3 nor IL-1*β* genes were modulated by* P. brasiliensis* infection. Thus, infected M-BMM from A/J preserves the profile associated with resistance (i.e., alternatively activated macrophages). Of note, GM-BMM from A/J showed similar pattern of NLRP3 and IL-1*β* expression when compared to M- and GM-BMM from susceptible B10.A mice (i.e., classically activated macrophages).

**Figure 4:**
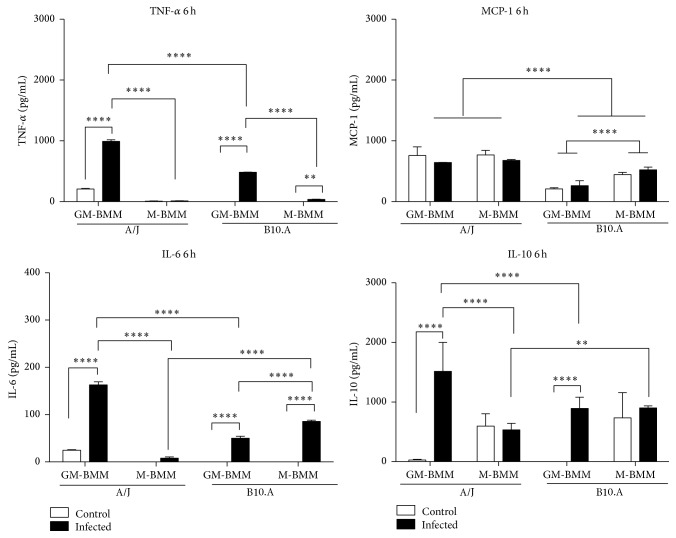
Cytokines profile after 6 h of* Paracoccidioides brasiliensis* infection of GM- and M-BMMs from A/J and B10.A mouse strains. Cytokines levels produced by the two different BMMs of both mouse strains after infection with Pb18, by ELISA assay. Data are means ± SD of triplicate samples representative of three separate experiments (^*∗∗∗∗*^ *p* ≤ 0.0001, ^*∗∗∗*^ *p* ≤ 0.001, and ^*∗∗*^ *p* ≤ 0.01).

**Figure 5:**
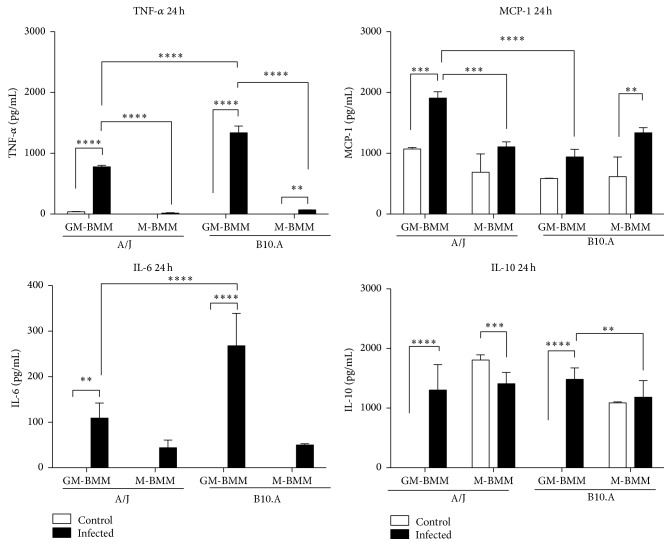
Cytokines profile after 24 h of* Paracoccidioides brasiliensis* infection of GM- and M-BMMs from A/J and B10.A mouse strains. Cytokines levels produced by the two different BMMs of both mouse strains after infection with Pb18, by ELISA assay. Data are means ± SD of triplicate samples representative of three separate experiments (^*∗∗∗∗*^ *p* ≤ 0.0001, ^*∗∗∗*^ *p* ≤ 0.001, and ^*∗∗*^ *p* ≤ 0.01).

Regarding the transcript levels of the cytokines IL-2, IL-6, IL-10, IL-12, and TNF-*α*, they were increased in GM-BMMs of both mice lineage when compared to M-BMMs. In fact, the protein levels of the proinflammatory cytokines IL-6 and TNF-*α* and the anti-inflammatory IL-10 were significantly increased, as assayed by ELISA at 6 and 24 h of infection (Figures 4 and 5), corroborating the transcript levels shown by the PCRarray. Interestingly, monocytes infected with the* P. brasiliensis* highly virulent isolate Pb18 produced higher levels of IL-6 and IL-10 than Pb265 isolate (low virulence) [52]. Thus, regardless of the mice strain, the isolate 18 of* P. brasiliensis* is induced in GM-BMMs to release into the environment context large amounts of both pro- and anti-inflammatory cytokines that may contribute to disturbances in immunity, which possibly leads to fungal survival. For instance, IL-6 production was associated to a significant increase in* P. brasiliensis* (Pb18 isolate) growth in monocytes [53]. Concerning IL-10, a large body of data exists, revealing the role of this regulatory cytokine with a compromised response to* P. brasiliensis* infection. In this sense, the simultaneous incubation of IL-10 with either IFN-*γ* or TNF-*α* inhibits murine macrophage fungicidal activity of these cells when cocultured with* P. brasiliensis*. The suppression of this activity by IL-10 was associated with the inhibition of nitric oxide production [54]. Furthermore, IL-10-knockout mice develop early T-cell responses, controlling the fungal growth in the lungs, resulting in an increased survival when compared to IL-10-sufficient mice [55].

**Figure 6:**
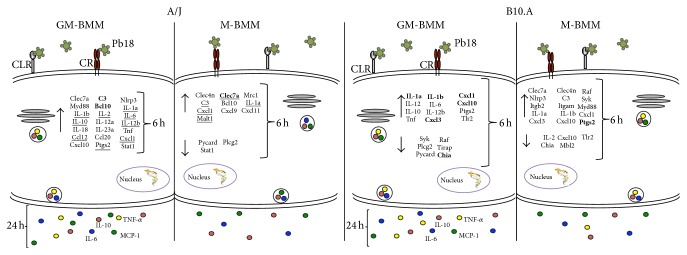
Schematic representation of the major results of gene expression and cytokine production by GM- and M-BMM from A/J and B10.A mouse strains in response to* in vitro* Pb18 infection. The arrows indicate up- and downregulated genes. The yellow, red, green, and blue circles are representative of TNF-*α*, IL-10, MCP-1, and IL-6, respectively. The genes indicated in bold demonstrated a higher expression compared between GM- and M-BMM from the same mouse strain. The underlined genes indicated the same expression pattern between GM- and M-BMM from different mice strain.

Altogether, our results as shown in Figure 6 demonstrate that GM-BMMs derived from A/J and B.10 produced higher levels of pro- and anti-inflammatory cytokines that may contribute to generate an unbalanced early immune response. It has been shown that the susceptible mouse lineage B10.A has an innate tendency to polarize into M1-like phenotype, whereas the opposite phenotype occurs in resistant A/J mice [31]. In this context, our data support that susceptibility and resistance are strongly associated with M1 and M2 polarization, respectively.

As final considerations, below we present some relevant and interesting data recently published in the field of innate immune regulation.

Since the development of high-throughput DNA/RNA sequencing methodologies, our understanding of transcriptional and posttranscriptional gene regulation increased at a never thought level [56, 57]. Furthermore, the advances in proteomic methods and the development of a myriad of innovative cell analysis techniques have also contributed to the better understanding of several highly complex biological processes in a more holistic view.

These approaches have been applied to detail the molecular basis involved in the innate immune response, tracing the path from the first molecular event, as PAMP-PRR interaction, to signalling cascades and transcriptional regulation, which in concert defines the control of pathogen-induced gene expression program [58–61]. In view of the importance of innate immune response to properly activate an inflammatory response to fight infection, its precise regulation is crucial to avoid host damage, as observed in several diseases.

Besides the huge amount of data revealing the importance of transcriptional regulation of the inflammatory genes expression, another equally important step of regulation, much less considered, operates at the posttranscriptional level [62, 63]. These authors stressed the role of alternative splicing, mRNA stability, and translational regulation, directly associated with components of the innate immunity. They presented several examples of differential splicing of TLR and signaling protein genes, resulting in functionally different isoforms, as well as the control at the level of mRNA stability and translation, providing a rapid and finely tuned response in its magnitude and extent. Altogether, these recent developments highlight the irrefutable importance of the coordination of regulatory mechanisms operating at the multiple layers of inflammatory genes expression, as a keystone in the control and modulation of host innate immune responses.

## Figures and Tables

**Table 1:** Comparison of differentially expressed genes in GM-BMM versus M-BMM of *Paracoccidioides brasiliensis* infected A/J and B10.A mice.

Gene	Name	Fold changes (FC)^*∗*^			
		A/J		B10.A	
		M-BMM + Pb18 versus M-BMM	GM-BMM + Pb18 versus GM-BMM	M-BMM + Pb18 versus M-BMM	GM-BMM + Pb18 versus GM-BMM
Pattern of recognition receptor (PRR)					
TLR2	Toll-like receptor 2	−1.7	1.4	**3.7**	**−4.3**
TLR4	Toll-like receptor 4	1.2	1.0	1.4	**−6.3**
TLR9	Toll-like receptor 9	−1.7	1.3	1.1	**−9.1**
Clec4n (Dectin-2)	C-type lectin domain family 4, member n	**6.4**	**2.1**	6.1	1.6
Clec7a (Dectin-1)	C-type lectin domain family 7, member a	**5.3**	**2.1**	**4.4**	1.1
Mrc1 (MR)	Mannose receptor, C-type 1	**3.0**	2.0	−1.7	−2.7
Nlrp3	NLR family, pyrin domain containing 3	1.3	**2.9**	**4.8**	−1.9
Scarf1	Scavenger receptor class F, member 1	**3.8**	−1.2	−1.7	−2.1
Itgb2	Integrin beta 2	−1.4	1.2	**2.7**	**−3.0**
Mbl2	Mannose-binding protein (protein C) 2	1.8	1.2	**−20.2**	**5.5**
CD14	CD14 antigen	2.0	3.0	−1.3	**8.8**
Itgam	Integrin alpha M	−1.4	1.6	1.3	**−3.6**
Colec12	Collectin subfamily member 12	**4.6**	1.8	**3.0**	−1.1
PRR signal Transduction					
Casp-8	Caspase-8	1.5	**3.9**	1.3	1.6
Irak4	Interleukin-1 receptor-associated kinase 4	1.3	**2.1**	1.5	−2.6
Mapk14	Mitogen-activated protein kinase 14	1.1	1.3	**2.3**	−3.6
Mapk8	Mitogen-activated protein kinase 8	−1.7	1.4	**2.0**	−1.6
MyD88	Myeloid differentiation primary response gene 88	1.5	**4.6**	**2.6**	−3.0
Bcl10	B-cell leukemia/lymphoma 10	**3.3**	**4.9**	1.6	2.0
Malt1	Mucosa associated lymphoid tissue lymphoma translocation gene 1	**2.4**	1.7	**3.1**	−1.7
Pycard	PYD and CARD domain containing	**−3.1**	1.3	1.7	**−5.5**
Plcg2	Phospholipase C gamma 2	**−2.2**	−1.1	2.0	**−4.9**
Tirap (Mal)	Toll-interleukin-1 receptor (TIR) domain-containing adaptor protein	−1.1	1.3	1.4	**−7.1**
Raf1	V-raf-leukemia viral oncogene 1	1.1	1.1	**3.2**	**−5.4**
Syk	Spleen tyrosine kinase	1.5	1.3	**2.9**	**−6.1**
CD40	CD40 antigen	−1.4	10.2	2.0	**10.9**
Transcription factor and other proteins					
Map2k4 (MKK4)	Mitogen-activated protein kinase 4	−1.3	1.8	1.9	**−4.5**
Mapk8	Mitogen-activated protein kinase 8	−1.7	1.4	**2.0**	−1.6
Nfkbia	Nuclear factor of kappa light polypeptide gene enhancer in B-cells inhibitor, alpha	−1.7	1.7	**2.0**	1.1
Cytokines					
Csf2 (GM-CSF)	Colony stimulating factor 2 (granulocyte-macrophage)	−3.3	3.1	−2.0	**−8.2**
Il1a	Interleukin-1 alpha	**5.4**	**15.0**	**4.5**	**5.8**
Il1b	Interleukin-1 beta	1.8	**31.4**	**3.4**	**7.0**
Il2	Interleukin-2	1.3	**124.7**	**−6.5**	**5.0**
Il6	Interleukin-6	1.0	**61.7**	−1.4	**4.4**
Il10	Interleukin-10	−1.8	**101.1**	1.5	**26.9**
Il12a	Interleukin-12A	1.3	**4.4**	1.8	3.5
Il12b	Interleukin-12B	1.5	**21.7**	1.5	**9.0**
Il18	Interleukin-18	−1.2	**2.4**	1.0	−1.3
Il23a	Interleukin-23, alpha subunit p19	1.2	**40.4**	2.0	1.9
Tnf	Tumor necrosis factor alpha	−1.0	**18.7**	−1.1	**18.6**
Chemokines					
Ccl5	Chemokine (C-C motif) ligand 5	1.1	6.2	3.5	21.1
Ccl12	Chemokine (C-C motif) ligand 12	−4.5	**6.9**	1.1	**7.7**
Ccl20	Chemokine (C-C motif) ligand 20	−1.1	**6.0**	**2.0**	1.0
Cxcl1 (KC)	Chemokine (C-X-C motif) ligand 1	**8.8**	**53.9**	**6.5**	**19.4**
Cxcl3	Chemokine (C-X-C motif) ligand 3	**3.5**	**3.4**	**15.2**	**64.4**
Cxcl9	Chemokine (C-X-C motif) ligand 9	**2.1**	**3.9**	1.5	3.7
Cxcl10 (IP-10)	Chemokine (C-X-C motif) ligand 10	−1.9	**6.8**	**2.5**	**8.9**
Cxcl11	Chemokine (C-X-C motif) ligand 11	**2.1**	−1.2	**−2.7**	−1.3
Other proteins					
Ptgs2 (Cox-2)	Prostaglandin-endoperoxide synthase 2	**8.3**	**28.0**	**18.8**	**11.3**
Chia1	Chitinase, acidic	−1.7	−2.0	**−3.4**	−4.9
Stat1	Signal transducer and activator of transcription 1	**−2.5**	**2.2**	−1.1	−2.6
C3	Complement component 3	**2.6**	**6.0**	**2.2**	1.1
C5ar1	Complement component 5a receptor 1	**3.4**	**2.7**	**2.8**	1.5
Fcgr3	Fc receptor, IgG, low affinity III	**4.9**	1.4	1.5	−1.3

^*∗*^Genes In bold lettering had their transcript levels significantly modulated (FC ≥ 2 or ≤ −2 and *p* value < 0.05 as described in Section 2). Positive and negative values represent genes with expression induced and repressed, respectively.
